# Prophylaxis of Childhood

**Published:** 1911-05-15

**Authors:** N. S. Hoff


### PROPHYLAXIS OF CHILDHOOD.

BY N. S. HOFF, D.D.S.

There can be little doubt that many infant disorders originate from the struggle which takes place as the teeth are developing and emerging from the gums, on account of the referred pain and the lack of normal function. Physicians have long recognized the cause and have resorted to all known medical expedient for relief, being most successful as a rule with remedies which control the central nervous functions, which every one recognizes as a most undesirable practice. The physiologic method should be found in careful scrutiny of the infant diet and oral functions, and much successful work has been accomplished along this line. The interest of the dental practitioner of oral prophylaxis is in the direction of improving dietary and functional conditions, not only for the infant health, but that a better development of the teeth and their oral environment may be secured.

Infant diet is one of the serious problems which confronts the mother, and necessarily so, since fully one third of all children born do not live over two years, and more than half of these deaths are traceable to diseases of the digestive function, mostly associated with the process of teething. It should not therefore seem inappropriate for the dentist to make some study of this problem, not only from the humanitarian standpoint, but also in the interest of good teeth.

For convenience in our study we shall classify childhood prophylaxis into three periods; first, infancy, or from birth to the beginning of first dentition, about eight months; secondly, the first dentition period, from eight months to six years; third, the period of permanent dentition or from the sixth to twelfth years. A fourth, or adolescent period might be added from twelve to twenty-one years; but as the teeth are practically formed prior to this period and mostly erupted there is not much to be studied in regard to systemic influences of a pathologic character so far as the teeth themselves are concerned.

**Infant Prophylaxis.** In certain social conditions of life it is estimated that thirty per cent of all children born die during the first week of their lives. The reason is obvious enough when it is realized in what conditions of poverty and unhygienic surroundings these children are born. The mothers are ignorant, unhealthy and careless as to personal habits and take no pains to provide themselves with proper medical attention or nursing, and the child does not receive much more care than the domestic animals born in the same homes. Undoubtedly a large number of such children would live if they could have proper nursing during the first two weeks of their lives. If so many children actually die from neglect of ordinary attentions to their bodies, what can we expect when there is total ignorance of the fact that any special care is needed which would favor a more healthy development of the tissues involved in the dental development? In good homes the newly born child is bathed and its eyes and mouth usually cleansed, but scarcely any nurse is bold enough to investigate the conditions of the nose and pharynx much less the passage to the lungs or stomach, to ascertain if any of the uterine fluids may have found their way into these passages to decompose there and seriously, if not fatally, infect the child. Of course the mucous linings of these passages are extremely sensitive and delicate and will not tolerate much interference, and yet we have no doubt that many a child would be made more comfortable, and perhaps its very life saved by some such inspection and treatment skillfully applied. It would at least be put into such condition that it could assimilate pure air and food, the two most important factors in its existence at this time. This means prophylactic treatment, which may not always be clearly indicated and yet it is so valuable, that little injury should result if applied even when not specially indicated.

Infants are especially susceptible to skin diseases and also diseases of the mucous membranes. These structures are exceedingly tender and if not carefully guarded are liable to eczema, intertrigo, laryningitis, bronchitis, etc., because of their exceeding susceptibility to irritation and local infection. All such diseases are preventable by absolute cleanliness and careful hygienic measures; and are not likely to occur in well born and hygienically cared for children, they are, however, liable to happen to any child because of accidents, inattention, bad nursing, or unwholesome feeding. Nurses are instructed to cleanse the new born child's mouth, but they should be warned that this may be done excessively and with permanent injury to the delicate organisms concerned in tooth formation. A suitable irrigation toilet of the mouth and nasal passages in early life would be of much benefit in warding off catarrhal troubles, adenoid development of the pharyngeal and faucial tonsils. Of course such treatments should only be administered by a competent nurse or physician using only a normal salt solution or a mild antiseptic lotion which would not irritate or interfere with normal mucous and glandular secretions. When we see the unwholesome conditions of the nasal passages in most infants we are inclined to wonder how there can ever be a normal development of the nasal and maxillary bones, and when we see children crawling along the dirty floors and putting everything they can pick up into their mouths, and inhaling the dust and dirt, we are certain a most kind Providence cares for children. We are not surprised that a long list of serious children's diseases await them with such certainty that it has become a fixed traditon to expect them as a matter inevitable and to pass them along from one member of the family to another. Such things are preventable by care, and because of their serious side influences and ultimate effects they should receive more careful attention in the preventive periods. All the nasal and oral maladies react through the nervous system on digestion and to a limited extent on the vital organs.

They not only predispose local malformations, or degenerative tendencies, but depress seriously mental functions, and so react forcibly on the general vitality of the body. The environment of the newly born child should be such as to promote good general health and to induce normal development of all structures. As nearly absolute cleanliness as circumstances will permit is fundamental, at the same time excessive bathing is exhausting, and the application of a cleansing toilet to the mucous tracts must be cautiously used. Fresh air and sunlight in an agreeable temperature are splendid stimulants to the proper development of the mucous membranes of the throat and lungs. The practice now very prevalent of keeping babies much out of doors is a helpful innovation. Nourishing foods, always considered the essential element in normal development, is of the utmost importance; but in these days of artificial feeding of babies we find that very grave errors are too often made in selecting a proper diet. A child that is fed on a chemically prepared food, which contains always the same identical chemical elements, will as truly starve as though it were fed on bread and water. It is impracticable to duplicate the mother's milk chemically. The child's appetite must be forced when it is artificially fed, and while all the food elements may be contained in such foods there is a lack of stimulation to the trophic nervous functions, so that food so taken is not digested and assimilated. Effective feeding therefore is mother's feeding, and it would seem logical to believe that herein is to be found the reason for the development of good teeth in the children of the poorer classes where artificial feeding does not extensively prevail. Undoubtedly there are many cases where the mother's milk is not sufficiently rich in some of the essential food elements. It is of course difficult to detect this in most cases until both the mother and child manifest it by a break in the normal condition. A skillful diagnostician who is accustomed to treating infants ought to be able to determine these conditions soon enough to prevent a climax, and to provide the materials lacking, preferably by changing the food or environment of the mother and child, or by artificial interference with the mother's diet, rather than to subject the child to medical treatment which would likely introduce digestive disturbances. If the aim should be to supply the nursing child with more lime salts for the teeth, it would be far better to increase the food containing these elements in the mother's dietary that her milk should be richer in them than to attempt to administer them to the child in artificial foods or as medicines, although this may sometimes become a necessary expedient. In many cases it is absolutely necessary to resort to artificial feeding entirely, as the mother is wholly incapable of feeding the child. The importance of securing a competent food in such cases can not be overestimated, in fact, it is one of the most serious problems connected with raising children. The most acceptable substitute food is cow's milk, and the difficulty of securing this in a pure and palatable form is very great, especially in cities where the milk of many cows is mixed together and it is kept for long periods in cold storage and is very liable to become seriously contaminated by almost innumerable handlings before it gets to the consumer. Too often this milk is adulterated or treated with antiseptics which are used to preserve it, and which are seriously harmful to the digestive processes. Several forms of condensed milk, milk flour, and medicated milks are used with some degree of success, but children obliged to depend upon such foods do not develop normally and we can not therefore expect the best degree of tooth calcification. Naturally we should expect faulty nourishment from long use of these artificial foods; in fact we not only have maldevelopment, but systemic diseases of the alimentary tract are common in children fed on artificial foods. If cows milk is used continuously the foreign albumins in cow's milk irritate the infants intestinal membrane and we have resulting what physicians have termed "cows milk disease." The effect of such artificial stimulation is that of final depression of the internal organs of nutrition to the point where they break down or rebel. Where babies are of necessity fed on cow's milk a slight modification has seemed advantageous; for instance, a small addition of lactose ($C12H22O11$), lime water &c., causes a material increase in calcium oxide, CaO, and phosphorus oxide, ($P2O5$). It is probably true that artificially fed babies receive a larger amount of food elements in prepared foods, but evidently they are incapable of assimilating all the food taken, and it remains in the stomach and intestinal canal to ferment and produce intestinal disturbances. Overfeeding is easy and consequently common where prepared foods are used. By careless or ignorant nurses children are overfed to prevent them from crying. Overfeeding is not most common with the poor, because they cannot afford the luxury, neither is it universally found in the families of the well to do and rich because they have capable nurses and medical advisors, consequently the very poor children are saved from many dietetic errors to which children born in somewhat better circumstances are liable because of thoughtlessness or ignorance. It is the great middle class that supports the medical profession, because this class lives a more generous and care free life, which sooner or later demands recompense for all unnatural and excessive drafts on the body, as well as the bank account. The middle class enjoys the present and lives excessively, the poor knows nothing better than to be content with frugal livng, while the rich and educated prize life and take precautions to preserve and extend it. This probably accounts for the small percentage of normal and typical dentitions of our time.

**Diseases of Children.** One of the most threatening diseases of childhood is tuberculosis. Few children are born with it, but contract it by absorption of its micro-organisms from external sources. Coughing, spitting, sneezing, and kissing should be barred from the nursery. Tubercular people should not live in contact with children or any other people; and the same can be said with regard to persons having such diseases as catarrh, diphtheria, measles, as all these diseases are readily contracted in infancy and will surely result in impaired development in oral and nasal organs and tissues. All eruptive diseases of the skin and mucous membrane are very likely to interrupt tooth calcification. Traumatic injuries to the mucous membrane should be carefully avoided, as they are likely to interrupt calcification and delay dentition. The slow development of the hard tissues and their nervous and vascular systems demand particular attention to induce normal growth. Rachitis is another disease closely associated with tooth formation. It is characterized by a lack of bone calcification and is ussually systemic; but it is not thought to be due to faulty nutrition, except as this may be caused by impaired nervous functions. The treatment advised would indicate that it should be regarded as atrophic. Pure air, even temperature, sunlight, cleanliness and a selected diet of wholesome food are advantageously used in its treatment. Its influence on the deciduous teeth will be indicated in poor calcification and early development of caries, delayed eruption and irregular alignment. It may be safely stated that all atrophic diseases and many of the nervous disorders of infancy will predispose to bad calcification of the deciduous teeth and they should receive preventive treatment with the fact in view.

**"Teething."** One of the most common and troublesome infantile diseases is teething. In respect to calcification of the deciduous teeth this malady is not of such importance to the dentist, as it is to the physician, because it exists whether the teeth are normally developed or not. Many medical writers maintain that it is a physiological process and it should not be considered pathologic. If it left no secondary systemic results it perhaps might be so considered, and such local disturbances as occur could be treated as local trauma. There can hardly be any question but teething produces a profound impression on the nervous system of the entire alimentary tract. In the mouth there is of course the local irritation, so severe at times as to cause excessive pain and some of the local symptoms of inflammation, but fortunately followed by none of the disastrous sequellae of suppurative inflammation. There is first an excessive salivary function from the reflex nervous irritations. The infant is taking little or no solid food requiring insalivation, consequently disordered digestion and intestinal functions result from swallowing quantities of saliva causing colic, diarrhoea and other children's complaints. Stomatitis of the oral mucous membrane and other severe local lesions result from these digestive disorders and because of the close contact of the oral surface tissues with the dental crypts and tooth follicles of the growing or forming teeth the calcifying processes are undoubtedly interrupted by both the local environment and the impaired nervous functions resulting in malformed and malposed teeth. Many children's diseases of digestion are attributed to bad or improper feeding and from reflected nerve impulses when their active cause can be traced to excessive salivary and mucous secretions. This excessive secretion passes into the stomach and intestinal tract where it prevents digestion, ferments and becomes a source of irritation and disease. The mouth and its associated sinuses and organs also become coated with abnormal adhesions which ferment and infect the purest food that can be provided for the child's nourishment, so that by the time it has passed to the digestive organs it is so contaminated that it can not be converted into assimilable food. The consequence is that the child must be relieved with a purge, and for lack of nourishment, experiences the greatest difficulty in recovering from such repeated shocks. The remedy evidently should be the disinfection of the mouth and intestinal tract and the removal or alleviation of the local irritating stimulants.

The practical disinfection of the mouth and intestinal tract for an infant is not easily accomplished. The mucous tissues are exceedingly delicate and easily injured by any mechanical or strong chemical reagent. It is possible to occasionally swab out a baby's mouth and throat with a soft sponge and a mild antiseptic solution, and the stomach's contents may be withdrawn with proper appliances skillfully used. Then the emerging teeth that are producing local irritations should receive such attention as would relieve the local congestion and the pain influence. There can be no good reason advanced in this day of aseptic surgery for not resorting to lancing of the gums when it promises such great relief and the chances for harm can be minimized. The pain of the operation is slight and can be somewhat avoided by a local application rubbed over the operating area. Proper after care of the wound in the way of cleanliness until resolution occurs is imperative. A child thus helped through the dentition period should develop a more normal and robust digestive organism and be saved much of the customary suffering of this period. Talbot calls attention to the trophic influence of the nervous system on first dentition, believing it to be a strong factor in diseases of this period. He regards these infantile maladies as symptomatic of neurotic degeneration. He warns against haemophilia which is a strong symptom of degeneration. Some form of oral antisepsis is required where children are artificially fed, as the food clings to the oral tissues and fauces, and by decomposing it becomes unwholesome. If a strong antiseptic wash is used or a weak one frequently there is danger of over stimulation and ulcerative infection. If the child could be taught to wash the mouth at necessary intervals with a warm solution of boric acid or normal salt solution this would be effective. Escherich suggests a pledget of cotton covered with linen or silk, on a suitable handle, which could be saturated with a sweetened solution of boric acid and which could be given to the child to chew upon at proper intervals as a possible method for keeping the mouth freed from infective materials. There can be little doubt that some preventive measures along this line would greatly mitigate infant suffering from digestive diseases and help to produce more uniform tooth calcification, with less maleruption of both the deciduous and permanent teeth.

